# An Unexpected Enzyme in Vascular Smooth Muscle Cells: Angiotensin II Upregulates Cholesterol-25-Hydroxylase Gene Expression

**DOI:** 10.3390/ijms24043968

**Published:** 2023-02-16

**Authors:** Kinga Bernadett Kovács, Laura Szalai, Pál Szabó, Janka Borbála Gém, Szilvia Barsi, Bence Szalai, Bernadett Perey-Simon, Gábor Turu, András Dávid Tóth, Péter Várnai, László Hunyady, András Balla

**Affiliations:** 1 Department of Physiology, Semmelweis University, 1094 Budapest, Hungary; 2 ELKH-SE Laboratory of Molecular Physiology, Eötvös Loránd Research Network and Semmelweis University, 1085 Budapest, Hungary; 3 Research Center for Natural Sciences, Center for Structural Study, MS Metabolomics Laboratory, 1117 Budapest, Hungary; 4 Research Centre for Natural Sciences, Institute of Enzymology, 1117 Budapest, Hungary; 5 Department of Internal Medicine and Haematology, Semmelweis University, 1088 Budapest, Hungary

**Keywords:** angiotensin II, atherosclerosis, cholesterol-25-hydroxylase, 25-hydroxycholesterol, vascular smooth muscle cell

## Abstract

Angiotensin II (AngII) is a vasoactive peptide hormone, which, under pathological conditions, contributes to the development of cardiovascular diseases. Oxysterols, including 25-hydroxycholesterol (25-HC), the product of cholesterol-25-hydroxylase (CH25H), also have detrimental effects on vascular health by affecting vascular smooth muscle cells (VSMCs). We investigated AngII-induced gene expression changes in VSMCs to explore whether AngII stimulus and 25-HC production have a connection in the vasculature. RNA-sequencing revealed that *Ch25h* is significantly upregulated in response to AngII stimulus. The *Ch25h* mRNA levels were elevated robustly (~50-fold) 1 h after AngII (100 nM) stimulation compared to baseline levels. Using inhibitors, we specified that the AngII-induced *Ch25h* upregulation is type 1 angiotensin II receptor- and G_q/11_ activity-dependent. Furthermore, p38 MAPK has a crucial role in the upregulation of *Ch25h*. We performed LC-MS/MS to identify 25-HC in the supernatant of AngII-stimulated VSMCs. In the supernatants, 25-HC concentration peaked 4 h after AngII stimulation. Our findings provide insight into the pathways mediating AngII-induced *Ch25h* upregulation. Our study elucidates a connection between AngII stimulus and 25-HC production in primary rat VSMCs. These results potentially lead to the identification and understanding of new mechanisms in the pathogenesis of vascular impairments.

## 1. Introduction

Cardiovascular disease (CVD) is the most common cause of death despite the decrease in CVD mortality throughout the years [1,2]. Atherosclerosis is one of the causative factors leading to the development of CVD. Both angiotensin II (AngII) and oxysterols are implicated in the pathological processes underlying atherosclerosis [3,4]. The present study aims to elucidate a connection between AngII and oxysterol production in primary vascular smooth muscle cells (VSMCs).

AngII is a vasoactive peptide hormone, which is the main effector molecule of the renin–angiotensin–aldosterone system (RAAS). Under physiological conditions, the RAAS regulates blood pressure through the alteration of blood volume and vascular resistance [5]. AngII exerts its physiological effects on VSMCs mainly through type 1 angiotensin II receptor (AT1R), which is a 7-transmembrane domain, G protein-coupled receptor (GPCR). The activated AT1R interacts with G_q/11_, G_12/13,_ and G_i_ heterotrimeric G proteins; hence, the activated signalization pathways upon ligand binding are diverse. The most characteristic is G_q/11_ signalization, which results in intracellular Ca^2+^ release and thus leads to VSMC contraction on tissue-level vasoconstriction [3]. It is well established that the AT1R signalization pathways in the vasculature are pleiotropic and involve growth factor receptor transactivation as well as the activation of numerous kinases [3]. The disrupted RAAS function, excessive AngII production, or AT1R activity promote pathological processes such as vascular remodeling [6]. AngII promotes reactive oxygen species (ROS) production, VSMC hypertrophy, proliferation and migration, collagen synthesis, the structural modification of vessel walls, and tumor necrosis factor-alpha (TNF-α) expression [7,8,9,10,11,12]. It is clear that AngII and AT1R signalization have serious implications for CVD. Yet, the exact signalization mechanisms and the induced cellular processes are not yet fully understood.

Oxysterols are the products of enzymatic or non-enzymatic reactions [13] and, similarly to AngII, can have detrimental effects on the vasculature [4,14,15]. For example, 25-hydroxycholesterol (25-HC) is the product of the cholesterol-25-hydroxylase (CH25H) enzyme, and it has several roles in various physiological functions. Indeed, 25-HC has been shown to possess a negative regulatory effect on cholesterol synthesis by inhibiting the proteolysis of sterol regulatory element-binding protein (SREBP) precursors, thus preventing the transcriptional events needed for cholesterol synthesis [16].

The oxysterol 25-HC is widely studied for its significant immunological properties, one of which is a strong antiviral action. It is exerted through the inhibition of viral entry, which has been described in the case of vesicular stomatitis virus (VSV), human immunodeficiency virus (HIV), Nipah virus (NiV), Ebola virus (EBOV), and Zika virus (ZIKV) [17,18]. Recently it was reported that 25-HC blocks the entry of severe acute respiratory syndrome coronavirus 2 (SARS-CoV-2), SARS-CoV, and Middle East respiratory syndrome coronavirus (MERS-CoV) [19]. CH25H and its oxysterol product are also mediators of innate immune responses. Following Toll-like receptor (TLR) agonist stimulus, CH25H induction and subsequent 25-HC production occur in dendritic cells and macrophages, which is regulated by type I interferons [20,21]. In vivo experiments revealed that TLR activation also results in elevated serum 25-HC levels in mice [20]. Oxysterols, including 25-HC, were found in the aortic tissue of hypercholesterolemic rabbits [22]. These hydroxylated products are also present in atherosclerotic plaques and are involved in atherosclerosis pathogenesis [14]. This is due to their role in promoting inflammatory cytokine production, foam cell formation, increasing matrix metalloproteinase-9 (MMP-9) expression, and contributing to vascular dysfunction through pro-oxidant effects [4,15,23]. In atherosclerotic lesions, macrophages express the chemokine interleukin-8 (IL-8), which is promoted by 25-HC in a dose-dependent and TLR-independent manner [24,25]. In macrophages, a proinflammatory response to 25-HC is triggered by the binding of 25-HC to integrins and the subsequent activation of focal adhesion kinase (FAK) signalization [26]. It was also demonstrated that 25-HC was able to induce ROS production in VSMCs [27]. The apoptotic effect of 25-HC on VSMCs has been described in primary cells derived from rabbits and chickens as well as in human VSMC cell lines [28,29,30,31]. It has been shown that 25-HC induces apoptosis via increased Ca^2+^ uptake of VSMCs [29] and, in addition, 25-HC promotes protein kinase A (PKA) dependent Bax phosphorylation and its subsequent translocation to the mitochondria, which leads to ROS production and the activation of the mitochondrial pathway of apoptosis [27]. VSMC apoptosis is notable in symptomatic plaques. It prominently occurs in the necrotic core and fibrous cap of atherosclerotic plaques; as a consequence, the fibrous cap grows thinner and, therefore, the risk of lesion rupture increases [32]. Another hallmark of atherosclerotic plaque formation is the phenotypic change and calcification of VSMCs, and these processes are also promoted by 25-HC [4,33].

Here, we present the novel findings that AngII stimulus markedly upregulates *Ch25h* expression through AT1R activation in rat primary VSMCs and, as a result of enzyme activity, 25-HC is present in the supernatant of cultured primary rat VSMCs. 

## 2. Results

### 2.1. AngII Induces Upregulation of Ch25h Gene Expression in Primary Rat VSMCs

**Figure 1:**
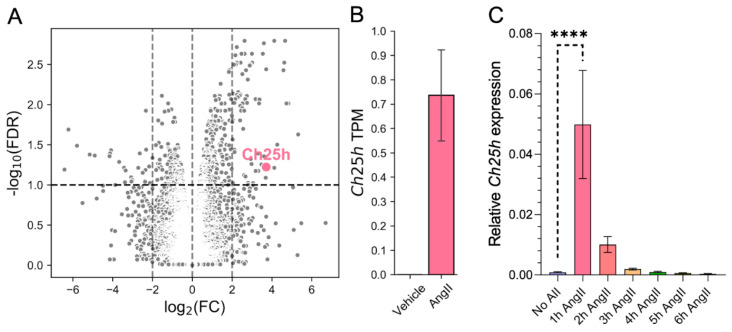
*Ch25h* gene expression is upregulated by AngII stimulus in rat VSMCs. (**A**) Serum-deprived VSMCs were stimulated with 100 nM AngII or treated with vehicle for 2 h. The results of the DE analysis of RNA-sequenced samples are shown as a volcano plot (x-axis: log_2_(FC, fold change) q, y-axis: −log_10_ (FDR, false discovery rate) based on the Benjamini–Hochberg correction of *p*-values. Of the significantly upregulated genes, the *Ch25h* is marked in pink. The horizontal line represents the significance threshold (FDR < 0.1). (**B**) The mean transcript per million (TPM) values of the *Ch25h* in 2 h AngII-stimulated (TPM = 0.74) VMSCs were changed compared to vehicle-treated (TPM = 0) VMSCs. (**C**) Serum-deprived VSMCs were stimulated with 100 nM AngII for 1, 2, 3, 4, 5, and 6 h or not stimulated. Total mRNA was isolated from these cells. Following cDNA preparation, qRT-PCR was performed. *Ch25h* mRNA levels are presented relative to *Gapdh*. Values are plotted as the mean ± SEM of n = 5 independent experiments. Data were analyzed using multiple linear regression, **** *p* < 0.0001.

To investigate transcriptomic changes associated with AngII-stimulation in rat VSMCs, we carried out RNA-sequencing (RNA-seq). Serum-deprived VSMCs were stimulated with 100 nM AngII or vehicle for 2 h. After preprocessing the RNA-seq data, we performed differential expression (DE) analysis (see Section 4). Considering the role of oxysterols and 25-HC in atherogenic processes [14,15], we were especially curious about how AngII affects *Ch25h* expression. *Ch25h*, which encodes the CH25H protein that catalyzes the formation of 25-HC, is significantly (*p*-value = 7.88 × 10^−4^, false discovery rate (FDR) corrected *p*-value < 0.1 based on Benjamini–Hochberg correction) upregulated in AngII stimulated samples. We found that *Ch25h* has the 10th highest log_2_FC among significantly (FDR < 0.1) upregulated genes (Figure 1A). *Ch25h* upregulation was significantly induced by AngII stimulation (Figure 1B), while no *Ch25h* mRNA was detected in vehicle-stimulated samples. The expression in AngII-stimulated samples increased to 0.74 TPM (Figure 1B).

To verify the RNA-seq results, we measured *Ch25h* mRNA levels in VSMCs using qRT-PCR. VSMCs were stimulated with 100 nM AngII for various time periods, namely 1, 2, 3, 4, 5, and 6 h or not stimulated. Our data show that *Ch25h* mRNA levels indeed increased in response to the AngII stimulus. *Ch25h* mRNA levels peaked 1 h after stimulus resulting in a more than fifty-fold increase compared to the baseline *Ch25h* mRNA levels (Figure 1C). Henceforth, we chose the 1 h stimulation time point to further analyze *Ch25h* expression characteristics in VSMCs.

### 2.2. AngII-Induced Ch25h Upregulation Is AT1R and G_q/11_ Activity-Dependent in Primary Rat VSMCs

**Figure 2:**
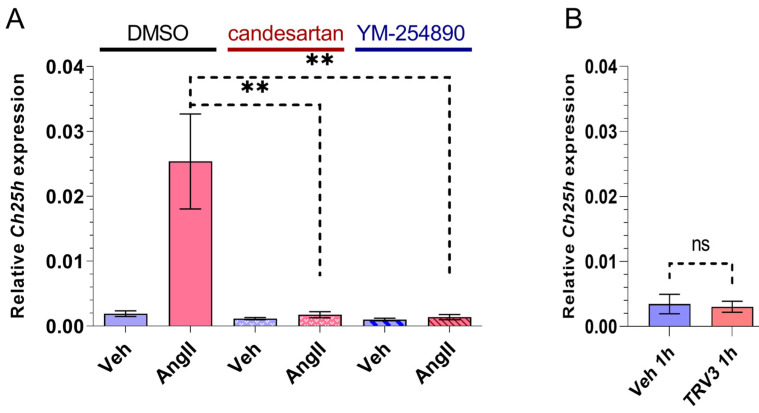
Effect of AT1R blocker, G_q/11_ inhibitor, and β-arrestin biased agonist on *Ch25h* gene expression in rat VSMCs. (**A**) Serum-deprived VSMCs were treated with 10 μM candesartan or 1 μM YM-254890 for 30 min, whereas the negative control group received DMSO treatment. Subsequently, VSMCs were stimulated with 100 nM AngII or vehicle (Veh) for 1 h. *Ch25h* mRNA levels were measured using qRT-PCR. *Ch25h* mRNA levels are presented relative to *Gapdh*. Values are plotted as the mean ± SEM of n = 4–5 independent experiments. Data were analyzed using multiple linear regression, ** *p* < 0.01. (**B**) Serum-deprived VSMCs were stimulated with 3 μM TRV120023 (TRV3) or vehicle (Veh) for 1 h. *Ch25h* mRNA levels are presented relative to *Gapdh*. Values are plotted as the mean ± SEM of n = 3 independent experiments. The unpaired *t*-test showed no significant changes in *Ch25h* expression between groups, (ns: not significant).

In order to determine the role of AT1R in *Ch25h* upregulation, we treated VSMCs with 10 μM candesartan AT1R antagonist for 30 min prior to 1 h 100 nM AngII stimulus. As expected, the candesartan pretreatment completely inhibited the AngII-induced *Ch25h* upregulation (Figure 2A). AT1R activation triggers signalization pathways typically through the G_q/11_ protein. To investigate the role of the G_q/11_ function in the upregulation of *Ch25h*, we utilized two approaches: the inhibition of the G_q/11_ pathway and the selective activation of the β-arrestin pathway. We pretreated the VSMCs with 1 µM YM-254890 G_q/11_ inhibitor for 30 min and then stimulated the cells with 100 nM AngII for 1 h. In a separate set of experiments, the VSMCs were stimulated with 3 µM TRV120023 (TRV3) peptide for one hour. TRV3 is a β-arrestin-biased AT1R agonist, and upon its receptor binding, the induced signalization does not include G_q/11_ protein activation [34,35,36,37]. Our qRT-PCR data indicate that YM-254890 pretreatment completely prevented *Ch25h* upregulation (Figure 2A), and TRV3 stimulus did not increase the mRNA level of *Ch25h* (Figure 2B).

### 2.3. Role of MAP Kinase Family Kinases in AngII-Induced Ch25h Upregulation in Primary Rat VSMCs

**Figure 3:**
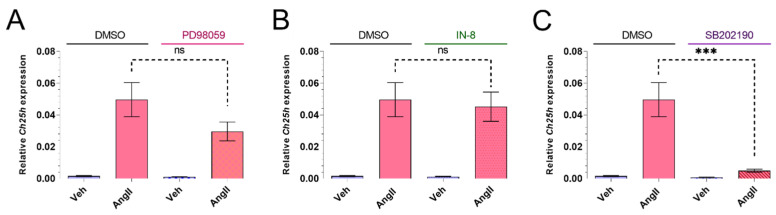
Effect of MAP kinase family inhibitors on *Ch25h* gene expression in rat VSMCs. (**A**) Serum-deprived VSMCs were treated with 20 μM PD98059, (**B**) 1 μM JNK-IN-8 (IN-8), or (**C**) 50 μM SB202190 for 30 min. The negative control group was treated with DMSO for 30 min in each experiment. Following treatment, VSMCs were stimulated with 100 nM AngII or vehicle (Veh) for 1 h. *Ch25h* mRNA levels are shown relative to *Gapdh*. Values are plotted as the mean ± SEM of n = 6 independent experiments. Data were analyzed using multiple linear regression, *** *p* < 0.001, ns (not significant).

Our data suggest that G_q/11_-mediated signaling pathways have a crucial role in *Ch25h* upregulation. MAP kinase family members such as ERK1/2, p38 mitogen-activated protein kinase (p38 MAPK), and c-Jun N-terminal kinase (JNK) are activated upon AT1R activation [3], mostly via G_q/11_ activation [38,39]. To explore the involved signaling events downstream of G_q/11_ protein activation, we used several MAPK family kinase inhibitors to assess the role of various MAPKs (Figure 3A–C). We used MEK inhibitor PD98059 (20 µM) for the elimination of ERK1/2 activity, SB202190 (50 µM) for p38 MAPK activity inhibition, and JNK-IN-8 (1 µM) to inhibit JNKs. VSMCs were pretreated for 30 min with one of the kinase inhibitors or DMSO as the control. Then, the VSMCs were stimulated with 100 nM AngII or vehicle for 1 h. *Ch25h* mRNA levels were assessed using qRT-PCR measurements. Figure 3A demonstrates that the PD98059 pretreatment caused slightly reduced AngII-induced *Ch25h* upregulation; however, this reduction was not significant. MEK and ERK1/2 activation might have some role in AngII-induced *Ch25h* upregulation, but this effect is not prominent. JNK-IN-8 (IN-8) pretreatment caused virtually no difference in *Ch25h* expression upon AngII stimulus compared to the DMSO-treated group (Figure 3B). In contrast, SB202190 pretreatment resulted in a significantly lower *Ch25h* expression in the AngII stimulated group (Figure 3C) compared to the DMSO control. Based on this result, it seems that p38 MAPK has a substantial role in AngII-induced *Ch25h* upregulation in VSMCs.

### 2.4. AngII-Induced Ch25h Upregulation Is Independent of NOX Activation in Primary Rat VSMCs

**Figure 4:**
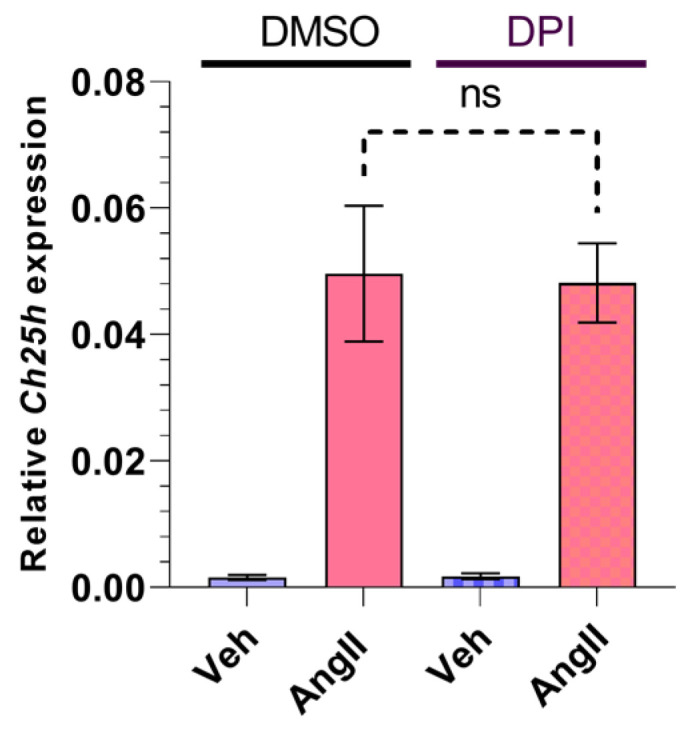
Effect of NOX inhibitor on *Ch25h* gene expression in rat VSMCs. Serum-deprived VSMCs were treated with 5 μM DPI or DMSO for 30 min. Following treatment, VSMCs were stimulated with 100 nM AngII or vehicle (Veh) for 1 h. *Ch25h* mRNA levels are shown relative to *Gapdh*. Values are plotted as the mean ± SEM of n = 5 independent experiments. Data were analyzed using multiple linear regression, ns (not significant).

AngII is able to induce NADPH oxidase (NOX) activity, which leads to increased ROS production [7,40]. Rat VSMCs express the NOX1 and NOX4 isoforms [40,41]. In order to investigate whether AngII-induced ROS production has any role in the subsequent upregulation of Ch25h, we used a diphenyleneiodonium chloride (DPI) pretreatment. DPI is a potent compound that inhibits the activity of NOX isoforms that are expressed in VSMCs [42]. We treated the VSMCs for 30 min with 5 µM DPI before the 1 h AngII (100 nM) stimulation. Our qRT-PCR measurements showed no significant difference between the DMSO- and DPI-treated groups (Figure 4).

### 2.5. Subcellular Localization of CH25H Protein in Rat VSMC Cell Line and Primary Rat VSMCs

**Figure 5:**
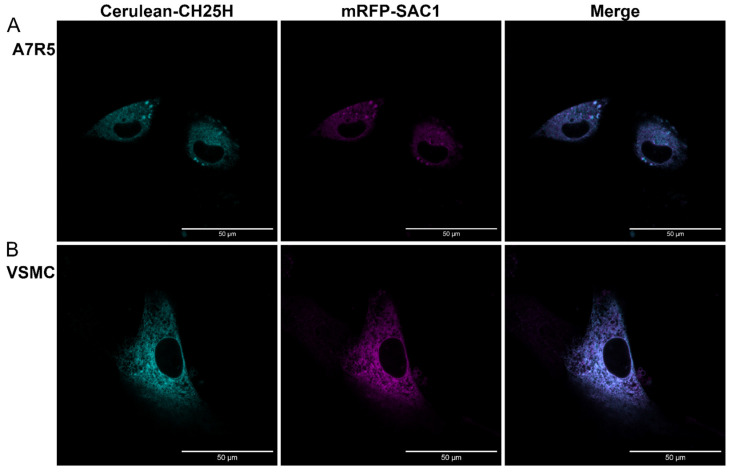
CH25H localizes to the endoplasmic reticulum in the A7R5 rat VSMC cell line and =primary rat VSMCs. (**A**) A7R5 cells and (**B**) primary rat VSMCs were cotransfected with DNA constructs encoding Cerulean-CH25H (cyan) and mRFP-SAC1 (magenta) fusion proteins. Twenty-four hours post-transfection, cells were examined using a Zeiss LSM710 confocal laser-scanning microscope. Merged images and colocalization analysis of signals show good colocalization of SAC1 endoplasmic reticulum marker and CH25H. Pearson’s correlation coefficient in A7R5 cells: 0.83 ± 0.038 = mean ± SEM, and in rat VSMCs: 0.85 ± 0.01 = mean ± SEM, n = 5 independent experiments. Fiji software was used for image processing and colocalization analysis. Scale bars represent 50 μm.

Based on the literature data, mouse and human CH25H proteins both localize to the endoplasmic reticulum (ER) and the Golgi compartment in transfected COS cells [16]. We investigated the subcellular localization of rat CH25H both in the A7R5 rat VSMC cell line (Figure 5A) and in primary rat VSMCs (Figure 5B) utilizing fluorescent proteins fused to either CH25H or an organelle marker protein. We created a DNA construct to transiently overexpress Cerulean-labeled CH25H fusion protein in cells alongside an mRFP-labeled phosphatidylinositol-3-phosphatase SAC1 (SAC1) fusion protein that localizes to the ER. Twenty-four hours after transfection, the cells were examined using confocal microscopy. Confocal images were analyzed with the JACoP plugin [43] to assess the colocalization of the two signals. The average values of Pearson’s correlation coefficient were 0.83 ± 0.038 and 0.85 ± 0.01 in the case of A7R5 and rat VSMCs, respectively. These Pearson’s correlation coefficient values indicate acceptable colocalization of signals. These results show that Cerulean-CH25H and mRFP-SAC1 colocalize, which confirms the ER localization of CH25H both in A7R5 cells and in primary VSMCs (Figure 5A,B). Additionally, we investigated the localization of Cerulean-CH25H to the Golgi using mRFP-TGN38 and mRFP-Giantin fusion proteins. Data obtained in these experiments show that CH25H and the Golgi markers do not colocalize (Supplementary Figure S2).

### 2.6. The Presence of 25-HC in VSMC Supernatant following AngII Stimulus

**Figure 6:**
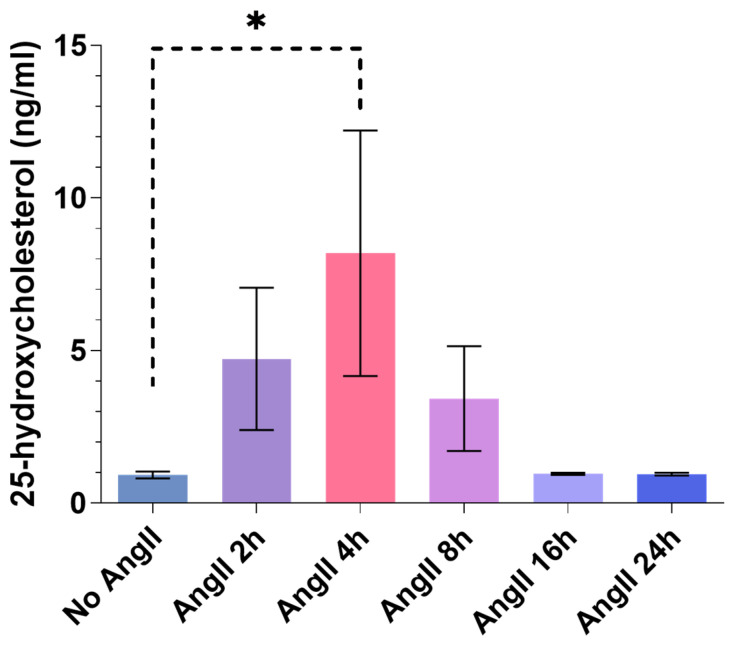
**The** 25-HC content of AngII-stimulated rat VSMC supernatant. Serum-deprived VSMCs were stimulated with 1 µM AngII for 2, 4, 8, 16, and 24 h or not stimulated. A total of 1 mL of supernatant of the cells was collected and subjected to LC-MS/MS analysis in order to identify 25-HC content and concentration. The 25-HC concentration is expressed in ng/mL. Concentration values are plotted as the mean ± SEM of n = 3 independent experiments. Data were analyzed using multiple linear regression, * *p* < 0.05.

It is known that 25-HC, the product of the CH25H enzyme, is capable of binding cell surface molecules and is able to induce various cell responses [26,27,33]; hence, it can function in the extracellular compartment. To find out whether VSMCs could be a source of extracellular 25-HC, we investigated the 25-HC levels in the supernatant of cultured rat VSMCs in response to AngII treatment (Figure 6). The examination of 25-HC levels also serves as an indicator of endogenous CH25H enzyme expression and activity in VSMCs. In order to detect 25-HC, the VSMCs were stimulated with 1 µM AngII for 2, 4, 8, 16, and 24 h or not stimulated. After the hormone stimulation, 1 mL of the supernatants was collected and subjected to LC-MS/MS measurement. The 25-HC concentrations were progressively increased reaching a peak at the 4 h time point (8.2 ng/mL on average) then returning to baseline levels 16 h post-AngII stimulation (Figure 6). We observed a significant, approximately 8-fold increase in the 25-HC level of VSMC supernatants 4 h after the AngII stimulus compared to the non-stimulated VSMC supernatants.

## 3. Discussion

In this study, we present the following findings: (1) AngII induces significant upregulation of *Ch25h* in primary rat VSMCs, (2) *Ch25h* upregulation is mediated by AT1R through G_q/11_ signaling and not via a β-arrestin-mediated mechanism, (3) p38 MAPK has a substantial role in *Ch25h* upregulation, (4) CH25H localizes to the ER in rat VSMCs, and (5) AngII stimulus promotes 25-HC production in primary rat VSMCs. Prior works show that AngII causes gene expression changes in various cell types [44,45,46,47]. Gene expression patterns shape cellular functions and are influenced by external stimuli [48]. Considering the role of AngII in the mediation of vascular remodeling [49] and its ability to activate diverse signalization pathways [3,50], it is crucial to better understand the long-term cellular responses induced by AngII. We examined gene expression changes promoted by AngII in rat primary VSMCs to clarify which genes are affected in response to AngII stimulus and to learn which pathways are involved in these gene expression changes.

In this paper, we show for the first time the upregulation of *Ch25h* expression by AngII in primary rat VSMCs. Our RNA-seq data shows significant upregulation of *Ch25h* in samples stimulated with AngII for 2 h compared to the vehicle-stimulated group (Figure 1A,B). According to our qRT-PCR measurements, *Ch25h* mRNA levels were the highest 1 h after the AngII stimulus, and 3 h after the AngII stimulus, *Ch25h* mRNA levels of the stimulated and non-stimulated groups were similar (Figure 1C). This result suggests that AngII-induced *Ch25h* upregulation is not sustained for longer periods of time in VSMCs. In contrast, various authors found that in different types of macrophages, the increase in *Ch25h* expression occurs 4 or 6 h post-stimulus [20,21,51]. In those cells, lipopolysaccharide (LPS) induces *Ch25h* upregulation in a type I IFN- and STAT1-dependent manner [21]. Taken together, these findings suggest that the *Ch25h* upregulation in VSMCs may be regulated in a different manner since *Ch25h* mRNA levels increase much quicker and transiently in VSMCs than in macrophages. This invites further study of *Ch25h* gene regulation in VSMCs.

We investigated the role of AT1R and different signalization pathways in the AngII-induced upregulation of *Ch25h* in VSMCs. AT1R activation is essential for the upregulation of *Ch25h*. This is demonstrated by the fact that the treatment of VSMCs with the AT1R antagonist candesartan completely wiped out the AngII-induced *Ch25h* mRNA level increase (Figure 2A). To distinguish between the various signalization pathways activated by AngII binding to AT1R, we used YM-254890 (a G_q/11_ protein inhibitor) and TRV120023 (a β-arrestin-biased agonist of AT1R) treatments. The inhibition of G_q/11_ obliterated *Ch25h* upregulation, whereas the TRV120023 treatment was not able to induce a *Ch25h* gene expression increase (Figure 2A,B). Based on these results, we conclude that *Ch25h* upregulation in response to AngII stimulus is exclusively AT1R and G_q/11_ activity-dependent in VSMCs.

G_q/11_ activation leads to inositol 1,4,5-trisphosphate (IP_3_) dependent Ca^2+^ release and diacylglycerol (DAG) production and, subsequently, ERK1/2 activation in cells [52]. Furthermore, it is well established that AngII stimulus can result in p38 MAPK and JNK activation [3]. This knowledge is especially interesting in light of one study by Bauman et al. where they demonstrated that the inhibition of p38 or JNK MAPK attenuated TLR4-mediated *Ch25h* mRNA increase in macrophages [20]. Considering the above-mentioned literature data, we examined the effects of SB202190, a p38 MAPK inhibitor, and JNK-IN-8, a JNK inhibitor. We found that SB202190 but not JNK-IN-8 inhibited the AngII-induced *Ch25h* upregulation (Figure 3B,C). These results indicated the role of p38 MAPK in *Ch25h* upregulation in VSMCs. In our experiments with the PD98059 MEK inhibitor, the AngII-induced *Ch25h* upregulation was somewhat affected by the inhibitor. However, the decrease in *Ch25h* mRNA levels in the PD98059-treated group was not significantly different from the DMSO-treated control cells (Figure 3A). This suggests that MEK and ERK1/2 activation are not essential in the upregulation of *Ch25h* expression in VSMCs, as the inhibition of these kinases was not effective to prevent *Ch25h* upregulation. Our findings point out that p38 MAPK signaling pathways are important in *Ch25h* gene expression regulation in VSMCs, but to fully understand their details further research is needed.

Based on the literature data we entertained the idea that NOX activation might play a role in AngII-induced *Ch25h* upregulation [13,53,54]. We found that DPI (a widely used NOX inhibitor) pretreatment did not have any effect on AngII-induced *Ch25h* mRNA increase. This suggests that NOX activity is not involved in the processes leading to *Ch25h* upregulation in VSMCs (Figure 4).

We examined the subcellular localization of CH25H in rat VSMCs. We found that the Cerulen-CH25H fusion protein and the mRFP-SAC1 ER marker fusion protein colocalized in both A7R5 cells and primary rat VSMCs (Figure 5A,B). This result shows that CH25H localizes to the ER both in the A7R5 VSMC cell line and in primary rat VSMCs, which is in line with previous information about the subcellular localization of the enzyme in other species [16].

The functional CH25H promotes the production of 25-HC which, by passing through the cell membrane, can act on several cell types both physiologically and pathologically [4,15,24,26,27]. Furthermore, 25-HC production is primarily the result of CH25H enzyme activity [13]. We measured 25-HC levels in the supernatant of AngII-stimulated primary rat VSMC cultures to determine whether the upregulation of *Ch25h* is associated with increased activity of the enzyme. Our results demonstrate that VSMCs indeed released 25-HC, the product of the CH25H enzyme, into their media (Figure 6). The 25-HC concentrations were at a peak 4 h after the AngII stimulation reached an average of 8.2 ng/mL. Following the 4 h time point, the 25-HC levels decreased, which may be due to its degradation or because of the reduced activity of CH25H over time. We observed a clear increase in supernatant 25-HC levels of cells stimulated with AngII for 2 and 4 h compared to non-stimulated cells. The 25-HC levels were decreasing from the 8 h time point onward. This trend is consistent in all of our experiments (Figure 6). Based on this result, we conclude that the observed 25-HC is the product of enzyme activity, and it is not generated by non-enzymatic reactions. This result shows that the CH25H enzyme is active in VSMCs.

Another notable finding is that 25-HC concentrations in VSMC supernatants varied in the ng/mL range. Studies involving 25-HC treatments usually utilize 25-HC concentrations ranging from 5 µg/mL to 50 µg/mL [27,28,29,30,31], which are thousand-fold higher concentrations than our measurements indicate. It is important to note that our 25-HC concentration data were obtained from VSMC supernatants of 1 mL volume. The released 25-HC dilutes under such conditions. In the interstitial compartments of the aortic vessel walls, the dilution would not be so prominent, meaning that the 25-HC concentration could be much higher in vivo. This allows 25-HC to be an efficient autocrine or paracrine mediator. Furthermore, 25-HC is able to directly bind cell surface proteins such as α5β1 and αvβ3 integrins [26]. α5β1 is expressed by VSMCs and is involved in processes of vascular injury [55]. Based on the findings of this study, we are eager to determine the effects of VSMC-produced 25-HC in the vasculature. Moreover, the possible functions of 25-HC intracellularly in VSMCs call for further investigation.

## 4. Materials and Methods

### 4.1. Materials

Cell culture plates were obtained from Greiner (Kremsmunster, Austria). μ-Slide 8 well plates were from Ibidi (Fitchburg, WI, USA). Reagents and biochemicals used during VSMC preparation were purchased from Duchefa Biochemie (Haarlem, The Netherlands) and Serva (Heidelberg, Germany). Collagenase type I was obtained from Worthington (Lakewood, NJ, USA). Dulbecco’s Modified Eagle Medium (DMEM) cell culture medium and fetal bovine serum (FBS) were supplied by Biosera (Nuaille, France). The Opti-MEM medium used during transfection procedures was purchased from Gibco (Dublin, Ireland). Penicillin–Streptomycin (Sigma-Aldrich, Darmstadt, Germany) and GlutaMAX (Gibco) were used to supplement the cell culture medium. Molecular biology reagents, RevertAid Reverse Transcription Kit, GeneJet Gel Extraction Kit, and the restriction and ligase enzymes were purchased from Thermo Fisher Scientific (Waltham, MA, USA). AngII and the inhibitors used in our study, namely: candesartan, SB202190, PD98059, and DPI were purchased from Sigma-Aldrich (St. Louis, MO, USA). JNK-IN-8 was purchased from Selleckchem (Houston, TX, USA). YM-254890 was obtained from Wako Chemicals (Neuss, Germany). TRV120023 (Sar-Arg-Val-Tyr-Lys-His-Pro-Ala-OH) peptide was synthesized by Proteogenix (Schiltigheim, France) to more than 98% purity. For total RNA isolation, Qiagen’s (Hilden; Germany) RNeasy Plus Mini kit was used. Quantitative real-time PCR (qRT-PCR) reactions were prepared using the SYBR Green Kit (LightCycler 480 SYBR Green I Master) from Roche (Basel, Switzerland). Primer oligos were synthesized by Sigma-Aldrich. Paraformaldehyde was from Polysciences (Warrington, PA, USA), other reagents, and the primer monoclonal anti-Actin, α-Smooth Muscle, clone 1A4 antibody used in immunocytochemistry were supplied by Sigma-Aldrich. Fluorophore-conjugated secondary antibodies and Lipofectamine 2000 transfection reagent were from Invitrogen (Carlsbad, CA, USA). FuGENE 6 transfection reagent was supplied by Promega (Madison, WI, USA). NEB10 competent *E. coli* was obtained from BioLabs (Ipswich, MA, USA). Unless otherwise stated, all other chemicals and reagents were purchased from Sigma-Aldrich Merck (St. Louis, MO, USA). The mRFP-SAC1 DNA construct was a kind gift from Dr. Péter Várnai (Semmelweis University).

### 4.2. Animals

Male Wistar rats (170–250 g, Charles River Laboratories-Semmelweis University, Budapest) were fed a standard semisynthetic diet. Our research conforms to the Guide for the Care and Use of Laboratory Animals (NIH, 8th edition, 2011) as well as national legal and institutional guidelines for animal care. This study was approved by the Animal Care Committee of Semmelweis University, Budapest, and by Hungarian authorities (No. 001/2139–4/2012). All procedures followed legal and institutional guidelines for animal care.

### 4.3. Isolation of Primary Rat VSMCs

VSMCs were isolated from 40–50 day old male Wistar rats weighing 170–250 g. VSMCs were prepared according to the standard explant method [56]. Briefly, animals were sacrificed by decapitation and fast bleeding. The thoracic aorta was excised and placed in a modified Krebs–Ringer solution (120 mM NaCl (Duchefa Biochemie); 4.7 mM KCl (Duchefa Biochemie); 1.8 mM CaCl_2_ (Duchefa Biochemie); 0.7mM MgSO_4_ (Fluka; Sleez; Hanover; Germany); 10 mM glucose (Serva); and 10 mM Na-HEPES (Serva). Following the removal of connective tissue and adherent fat, the aorta was cut into 1 mm sections, and aorta-rings were digested with collagenase (Collagenase type I., Worthington) for 25 min at 37 °C. VSMCs were allowed to grow onto 10 cm cell culture plates from the explants for 7–14 days incubated at 5% CO_2_ and 37 °C. VSMCs were cultured in Dulbecco’s modified Eagle media (DMEM High Glucose W/ L-Glutamine W/Sodium Pyruvate; Biosera) containing 10% fetal bovine serum (FBS; Biosera), 1% penicillin–streptomycin (Sigma-Aldrich), and 1% GlutaMAX (Gibco). The VSMCs were passaged with trypsin (EuroClone, Milan, Italy) and were used between passages 2 and 3. Typically, the experiments were performed at the third passage. The expression of smooth muscle α-actin was confirmed using immunochemistry.

### 4.4. Next-Generation RNA Sequencing

Primary rat VSMCs were serum deprived overnight prior to hormone stimulus. VSMCs were stimulated with 100 nM AngII for 2 h at 37 °C. VSMCs were washed twice with cold sterile PBS (137 mM NaCl; 2.7 mM KCl 2.7; 10.1 mM Na_2_HPO_4_; 1.8 mM KH_2_PO_4_, pH 7.4). Cell lysis was carried out using Trizol reagent (Thermo Fisher Scientific, Waltham, MA, USA). Total RNA isolation and next-generation RNA sequencing were performed using UD-GenoMed Medical Genomic Technologies Ltd., University of Debrecen, Debrecen, Hungary.

### 4.5. Differential Expression Analysis

As a result of RNA-sequencing, raw data were obtained in the fastq format. The data were processed using kallisto v0.46.2, a program for quantifying the abundance of reads with high accuracy [57]. The quantification is obtained in transcripts per million (TPM) and estimated counts. Ensembl Rnor_6.0 reference transcriptome was used for indexing. From transcript level abundances, differential expression between control and stimulated samples were calculated using the voom, lmFit, and eBayes functions of the *limma* R package v3.50.1 [58].

### 4.6. Inhibitor Treatments and Hormone Stimulation of VSMCs

Before the experiments, the VSMCs were serum deprived overnight using DMEM supplemented with 0.1% bovine serum albumin (BSA; Sigma-Aldrich). VSMCs were pretreated with either dimethyl sulfoxide (DMSO) vehicle or various inhibitors separately: 10 µM candesartan, 1 µM JNK-IN-8 (IN-8), 50 µM SB202190, 20 µM PD98059 (Sigma-Aldrich), 1 µM YM-254890 (Wako Chemicals), and 5 µM DPI (Sigma-Aldrich). Pretreatment lasted for 30 min. Following pretreatment, VSMCs were stimulated with 100 nM AngII or vehicle for 1 h. In the other sets of experiments, the VSMCs were stimulated with either vehicle or 3 µM TRV120023 (TRV3; Proteogenix) for 1 h. To assess time dependency of *Ch25h* mRNA expression, VSMCs were stimulated with 100 nM AngII for 1, 2, 3, 4, 5, and 6 h or not stimulated.

For the detection of 25-HC, VSMCs were stimulated with 1 µM AngII for 2, 4, 8, 16, and 24 h or not stimulated. The experiments were performed in duplicates. After the hormone stimulation, the supernatants were collected and subjected to LC-MS/MS measurement.

### 4.7. RNA Isolation from VSMCs and cDNA Preparation

VSMCs were washed twice with cold, sterile PBS, and the total RNA was isolated using the RNeasy Plus Mini kit from (Qiagen). RNA concentrations were determined with spectrophotometry at 260 nm, and purity was assessed using the 260/280 and 230/260 nm ratios. For cDNA preparation, 0.1 µg/µL total RNA dilution was used. Reverse transcription was carried out with the RevertAid Reverse Transcription Kit (Thermo Fisher Scientific) according to manufacturer’s instructions.

### 4.8. Quantitative Real-Time PCR (qRT-PCR)

We quantified mRNA levels using quantitative real-time PCR (qRT-PCR). qRT-PCR reactions were prepared using SYBR Green Kit (LightCycler 480 SYBR Green I Master; Roche) according to manufacturer’s instructions. The measurements were carried out with the LightCycler 480 instrument (Roche). We assessed target gene expression levels relative to the glyceraldehyde-3-phosphate dehydrogenase (*Gapdh*) mRNA level. The following primers were used for qRT-PCR determinations: *Gapdh*: Forward: 5′ CCTGCACCACCAACTGCTTAG 3′, Reverse 5′CAGTCTTCTGAGTGGCAGTGATG 3′; *Ch25h*: Forward: 5′ GCGTTGGCTACCCAATACAT 3′; Reverse: 5′ GTGAGTGGACCACGGAAAGT 3′. The thermal cycling program was as follows: pre-incubation starts at 95 °C for 5 min, followed by amplification 45 cycles of 10 s at 95 °C, 5 s at 62 °C, and 15 s at 72 °C, melting curve 5 s at 95 °C, 1 min at 65 °C and 97 °C, and cooling 30 s at 40 °C. Fluorescence data including melting curves were obtained. The cycle threshold (Ct) was calculated with the second derivative method using LightCycler 480 Software. ∆Ct is the difference in Ct values obtained between the reference and the tested samples. Fold ratios of gene expression were calculated as follows: Ratio = E ^∆Ct target gene^/E ^∆Ct GAPDH^.

### 4.9. DNA Constructs

We constructed DNA plasmids to express fluorescently tagged CH25H protein. We created a Cerulean-labeled plasmid construct using the backbones of pEYFP-N1-Cerulean (Clontech, Mountain View, CA). In order to amplify the entire *Ch25h* ORF region, the cDNA sample of VSMCs stimulated with AngII for 1 h was used as a template. The following primers were used during PCR amplification: forward 5′ ATATATGGCCTGCCACAACGTTTCG 3′; reverse 5′ ATATAGTCTGTTTCTTCTTCTGGTTCAAGTG 3′. The PCR product was electrophoresed, purified using the GeneJet Gel Extraction Kit (Thermo Fisher Scientific), and subjected to a second round of PCR amplification with primers containing restriction enzyme sites. Forward primer containing EcoRI site: 5′ ATATGAATTCGCCACCATGGCCTGCCACAACGTTTCG 3′. Reverse primer containing AgeI site: 5′ ATATACCGGTCTGTTTCTTCTTCTGGTTCAAG 3′. This PCR product and the pEYFP-N1-Cerulean backbone were then digested with EcoRI and AgeI restriction enzymes (Thermo Fisher Scientific) according to manufacturer’s instructions. *Ch25h* insert and pEYFP-N1-Cerulean were then incubated overnight at 16 °C with T4 ligase and T4 ligase buffer (Thermo Fisher Scientific). The completed Cerulean-CH25H plasmid construct was cloned in NEB10 bacteria (BioLabs).

### 4.10. Transfection

A7R5 rat aortic VSMCs (American Type Culture Collection, Rockville, MD, USA) were plated onto μ-Slide 8 well plate (Ibidi) in a 1 × 10^5^ cells/well density. The next day, A7R5 cells were cotransfected with Cerulean-CH25H and mRFP-SAC1 fusion protein expressing DNA constructs (0.15 μg DNA/well) using Lipofectamine 2000 transfection reagent and Opti-MEM (Gibco) according to manufacturer’s instructions. In a separate set of experiments, A7R5 cells were cotransfected with Cerulean-CH25H and mRFP-TGN38 or Cerulean-CH25H and mRFP-Giantin fusion protein expressing DNA constructs using the transfection protocol described above.

In the case of primary rat VSMCs, the cells were plated onto μ-Slide 8 well plate in a 1 × 10^5^ cells/well density. Transfection took place the following day. VSMCs were cotransfected with Cerulean-CH25H and mRFP-SAC1 constructs (0.5 μg DNA/well) using FuGENE 6 transfection reagent (Promega) applying a 3:1 = FuGENE 6:DNA ratio. We followed the manufacturer’s instructions during the transfection procedure.

### 4.11. Immunocytochemistry

To assess the homogeneity and purity of the primary rat VSMC cultures used in our experiments, smooth muscle alpha-actin was labeled (Supplementary Figure S1). Cells were washed with cold, sterile PBS and then fixed using 4% paraformaldehyde (PFA; Polysciences) for 15 min. Following fixation, VSMCs were permeabilized with 0.1% Triton X-100 for 5 min then incubated in 0.1% sodium–borohydride solution for 15 min. VSMCs were incubated for 30 min in a blocking solution containing 1% bovine serum albumin (Sigma-Aldrich). Immunolabeling took place with anti-smooth muscle alpha-actin monoclonal mouse primary antibody (A2547; Sigma-Aldrich) alongside Alexa Fluor 488-conjugated anti-Mouse IgG secondary antibody. Between each step, cells were washed three times with PBS. To label cell nuclei, TO-PRO3 nucleic acid stain was used (Thermo Fisher Scientific).

### 4.12. Microscopy

For the imaging of fluorescently labeled smooth muscle alpha-actin in rat VSMC samples and the fluorescent signal of fusion protein expressing cotransfected A7R5 and rat VSMC samples, a Zeiss LSM 710 (Oberkochen, Germany) confocal laser-scanning microscope was used. Imaging of the transfected cells was carried out 24 h after cotransfection. The obtained images were processed with Fiji 1.53q software [59]. Colocalization analysis was carried out using the JACoP plugin, and Pearson’s correlation coefficient values were used to define colocalization [43].

### 4.13. Liquid Chromatography–Tandem Mass Spectrometry (LC-MS/MS)

A total of 900 µL of methanol was used for 300 µL of supernatant samples for protein precipitation. The supernatants were obtained from 6-well plates, in which the cells were cultured in 1 mL of medium. The samples were vortexed and centrifuged for 5 min at 13,000 rpm. A total of 100 µL of supernatant was pipetted into a micro-vial and used for quantitation. LC-MS/MS measurements were run on a Sciex 6500QTrap mass spectrometer coupled with an Agilent 1100 HPLC system. A Kinetex EVO-C18, 50 × 2.1 mm, 5 µm HPLC column was applied using water and acetonitrile (both containing 0.1% formic acid) in that gradient elution mode. The flow rate was 600 µL/min. The mass spectrometer was operated in positive APCI ionization. The needle current was set to 3 µA. Curtain, evaporating, and drying gases were 40, 30, and 20 psi, respectively. Quantitation was completed in the MRM mode using ion transitions: 382.5/367.2, 367.2/161.3, 385.2/159, and 367.3/159. The dwell time for each transition was 150 msec.

### 4.14. Statistical Analysis

Statistical analysis and graph plotting were carried out using GraphPad Prism 9.1.2 (San Diego, CA, USA) software. The sample size is given in the figure legends as n = the number of independent experiments. Data are shown as mean ± SEM. Gene expression data obtained from qRT-PCR measurements were analyzed using multiple linear regression with a 95% confidence interval in order to determine the significance of inhibitor treatments, stimuli, and their interaction with the dependent variable. The 25-HC concentration values were analyzed using multiple linear regression. In the case of data displayed in Figure 2B, an unpaired *t*-test was used to compare the control and the stimulated group.

## 5. Conclusions

In this study, we report that AngII promoted the upregulation of *Ch25h* in VSMCs. *Ch25h* expression in VSMCs was dependent on AT1R and subsequent G_q/11_ activation. Our experiments using various inhibitors showed the substantial role of p38 MAPK in *Ch25h* upregulation induced by AngII. CH25H localized to the ER of VSMCs. Our data demonstrated that 25-HC concentration was elevated in the supernatants of AngII-stimulated VSMCs, meaning that the CH25H enzyme was active in VSMCs. Our work elucidates the effect of AngII on gene expression changes in VSMCs and invites further studies of CH25H—an enzyme having primarily immunological functions—in the context of the vasculature.

## Data Availability

The datasets generated during the current study are available from the corresponding authors on reasonable request. RNA-seq data have been deposited in the ArrayExpress database at EMBL-EBI www.ebi.ac.uk/arrayexpress (accessed on 3 July 2022) under accession number E-MTAB-11863.

## Supplementary Materials

The following supporting information can be downloaded at: https://www.mdpi.com/article/10.3390/ijms24043968/s1.
