# Supplementary material for: An Unexpected Enzyme in Vascular Smooth Muscle Cells: Angiotensin II Upregulates Cholesterol-25-Hydroxylase Gene Expression
Source: Int J Mol Sci. 2023 Feb 16;24(4):3968. doi: 10.3390/ijms24043968 (PMC9965395; doi:10.3390/ijms24043968)
Supplement: Supplementary file 1 [file ijms-24-03968-s001.zip › ijms-2187929-supplementary.pdf]

Supplementary Material

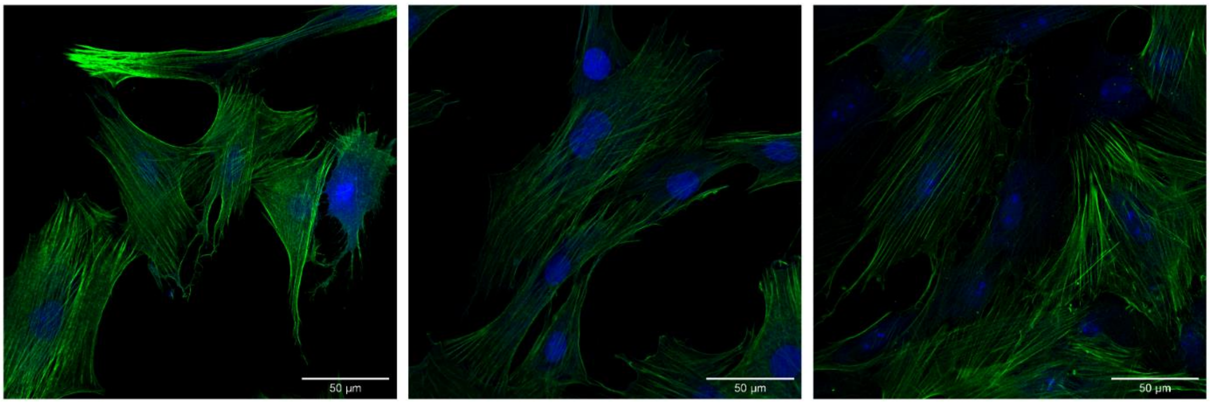

Supplementary Figure S1. Verification of primary rat VSMC culture homogeneity. Primary rat VSMCs were fixed with 4% PFA solution, and permeabilized with 0.1% Triton-X solution. Smooth muscle alpha actin (green) was labeled with monoclonal mouse antibody. Cell nuclei (blue) were stained with TO-PRO3 nucleic acid stain. VSMCs were imaged with Zeiss LSM 710 confocal laser-scanning microscope. Fiji software was used for image processing. Scale bars represent 50 µm.

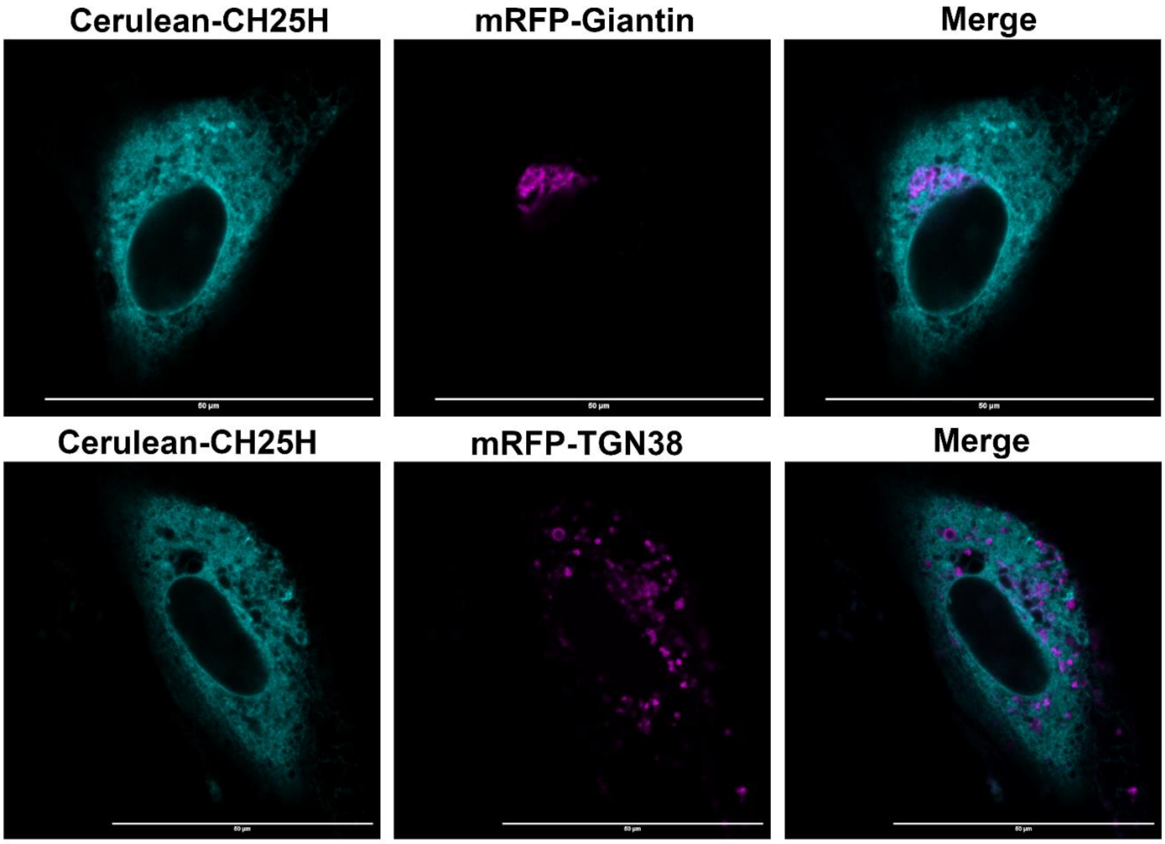

Supplementary Figure S2. CH25H does not show colocalization with Golgi markers. A7R5 cells were cotransfected with DNA constructs encoding Cerulean-CH25H (cyan) and mRFP-Giantin - marker of Golgi apparatus membrane - or mRFP-TGN38 – marker of trans-Golgi network membrane - (magenta) fusion protein. 24 hours post-transfection, cells were examined using Zeiss LSM710 confocal laser-scanning microscope. Merged images and colocalization analysis of signals show no colocalization of either Golgi marker protein and CH25H. Pearson's correlation coefficient in case of Cerulean-CH25H and mRFP-Giantin signals: 0.29±0.014 = mean±SEM and in case of Cerulean-CH25H and mRFP-TGN38: 0.41±0.013 = mean±SEM, n = 3 independent experiments. Fiji software was used for image processing and colocalization analysis. Scale bars represent 50 µm.
